# Serum Zinc Level Classification System: Usefulness in Patients with Liver Cirrhosis

**DOI:** 10.3390/jcm8122057

**Published:** 2019-11-22

**Authors:** Hiroki Nishikawa, Hirayuki Enomoto, Kazunori Yoh, Yoshinori Iwata, Yoshiyuki Sakai, Kyohei Kishino, Naoto Ikeda, Tomoyuki Takashima, Nobuhiro Aizawa, Ryo Takata, Kunihiro Hasegawa, Noriko Ishii, Yukihisa Yuri, Takashi Nishimura, Hiroko Iijima, Shuhei Nishiguchi

**Affiliations:** 1 Division of Hepatobiliary and Pancreatic disease, Department of Internal Medicine, Hyogo College of Medicine, Nishinomiya, Hyogo 663-8501, Japan; enomoto@hyo-med.ac.jp (H.E.); mm2wintwin@ybb.ne.jp (K.Y.); yo-iwata@hyo-med.ac.jp (Y.I.); sakai429@hyo-med.ac.jp (Y.S.); hcm.kyohei@gmail.com (K.K.); nikeneko@hyo-med.ac.jp (N.I.); tomo0204@yahoo.co.jp (T.T.); nobu23hiro@yahoo.co.jp (N.A.); chano_chano_rt@yahoo.co.jp (R.T.); hiro.red1230@gmail.com (K.H.); ishinori1985@yahoo.co.jp (N.I.); gyma27ijo04td@gmail.com (Y.Y.); tk-nishimura@hyo-med.ac.jp (T.N.); hiroko-i@hyo-med.ac.jp (H.I.); nishiguc@hyo-med.ac.jp (S.N.); 2 Center for clinical research and education, Hyogo College of Medicine, Nishinomiya, Hyogo 663-8501, Japan

**Keywords:** serum zinc, classification system, liver cirrhosis, Child–Pugh classification, ALBI grade, prognosis

## Abstract

Currently, the Japanese society of clinical nutrition (JSCN) defines serum zinc (Zn) level < 60 μg/dL as Zn deficiency and 60 μg/dL ≤ serum Zn level < 80 μg/dL as subclinical Zn deficiency, and 80 μg/dL ≤ serum Zn level < 130 μg/dL as normal Zn range. We aimed to elucidate the prognostic impact of this Zn classification system in patients with liver cirrhosis (LC) compared to the Child–Pugh classification and the albumin–bilirubin (ALBI) grading system (*n* = 441, median age = 66 years). The Akaike information criterion (AIC) with each evaluation method was tested in order to compare the overall survival (OS). The median serum Zn level was 65 μg/dL. There were 56 patients with normal Zn level, 227 with subclinical Zn deficiency and 158 with Zn deficiency. OS was well stratified among three groups of serum Zn level (*p* < 0.0001). The AIC value for survival by the Zn classification system was the lowest among three prognostic models (AIC: 518.99 in the Child–Pugh classification, 502.411 in ALBI grade and 482.762 in the Zn classification system). Multivariate analyses of factors associated with OS revealed that serum Zn classification by JSCN was an independent factor. In conclusion, the serum Zn classification proposed by JSCN appears to be helpful for estimating prognosis in LC patients.

## 1. Introduction

Liver cirrhosis (LC) is a terminal form of chronic liver damage. It is often accompanied by several clinical manifestations including hepatic encephalopathy (HE), ascites, varices caused by portal hypertension or liver carcinogenesis, with all of them leading to dismal prognosis [1,2,3,4]. The Child–Pugh (C–P) classification system is popular among hepatologists for the evaluation of hepatic function in patients with LC [5]. However, the major limitation of this system is that it includes several subjective components (hepatic encephalopathy and ascites) and interrelated components (serum albumin and ascites) [5]. To overcome these obstacles, a simple grading system for the evaluation of hepatic function, called the albumin–bilirubin (ALBI) grade, which is calculated by only two serum markers (i.e., serum albumin level and total bilirubin level), has been recently reported [6]. The well predictability of the ALBI grading system has been confirmed in LC patients with or without hepatocellular carcinoma (HCC) [7,8,9,10,11,12,13,14]. Recently, the modified ALBI grade using an additional cut-off value for the ALBI score has been reported as a useful predictor for patients with HCC [15]. 

Zinc (Zn) is an essential trace element for normal cell development, proliferation and differentiation, and its deficiency can cause abnormal taste, dermatitis, hair loss, anemia, stomatitis, male sexual dysfunction, susceptibility to infection, and osteoporosis [16,17,18,19]. Zn is widely distributed in the human body and the most abundant Zn is in muscle (60%), followed by bone (20–30%), skin and hair (8%), and liver (4–6%) [20]. In many patients with LC, diabetes, chronic inflammatory bowel diseases, and chronic kidney diseases, serum Zn levels have been reduced, indicating that they are in a state of Zn deficiency [19,21,22,23]. Hypozincemia (serum Zn level <55 μg/dL) has been linked to liver carcinogenesis in patients with hepatitis C virus (HCV)-related LC [24]. Oral Zn supplementation therapy can ameliorate LC-related HE [19,25]. Serum Zn level can also be associated with sarcopenia as defined by muscle mass decline and muscle strength decline in chronic liver diseases [18].

Currently, the Japanese society of clinical nutrition (JSCN) defines serum Zn level < 60 μg/dL as Zn deficiency and 60 μg/dL ≤ serum Zn level < 80 μg/dL as subclinical Zn deficiency, and 80 μg/dL ≤ serum Zn level < 130 μg/dL as normal Zn range (http://www.jscn.gr.jp/pdf/aen20180402.pdf). However, the prognostic impact of this classification system for serum Zn level in patients with LC remains unclear. Additionally, the relationship between this classification system and clinical markers have not been fully examined in patients with LC. In this study, we attempted to clarify these important research problems. 

## 2. Patients and Methods

### 2.1. Patients

Five hundred and sixty-eight LC individuals for whom data on baseline serum Zn level were available were admitted to our institution between October 2005 and June 2018. Twenty-eight patients unfollowable within one year were excluded from the analysis. In the remaining 540 patients, 99 patients with the confirmation of HCC on imaging findings at baseline or those with a past history for HCC were excluded. A total of 441 LC subjects were thus analyzed in this analysis. 

In the observation period, blood tests and radiological tests for the purpose of detecting HCC occurrence or LC-related complications were performed every 3 to 6 month interval. Determination of LC was based on pathological data, imaging findings such as presence of splenomegaly or varices and/or laboratory data such as hypoalbuminemia or prolongation of prothrombin time (PT) [26,27,28]. There were 261 patients (59.2%) with histologically proven LC. When serum albumin level decreased to less than 3.5 g/dL during the follow-up period, nutritional therapies such as branched-chain amino acid therapies were in consideration [29,30]. In cases with HCV or hepatitis B virus (HBV), antiviral treatments (i.e., direct acting antivirals (DAAs), interferon-based therapies or nucleoside analogues (NAs)) were also in consideration [29]. In cases with hypozincemia (serum Zn level <60 μg/dL in our hospital), we considered Zn supplementation therapy such as polaprezinc or Zn acetate hydrate according to the recommendations of the manufacturer. Principally, diagnosis and treatment strategies for HCC were determined based on the current standard guidelines [31,32].

### 2.2. ALBI Score and ALBI Grade

The calculation of the ALBI score was: ALBI score = (log 10 total bilirubin [μmol/L] × 0.66) + (serum albumin [g/L] × –0.085), while the classification of the ALBI grade was: ALBI score ≤ –2.60 = grade 1, –2.60< ALBI score ≤ –1.39 = grade 2 and ALBI score > –1.39 = grade 3 [6]. 

### 2.3. Serum Zn Classification and Our Study

Our current study was a retrospective observational study. Patients with 80 μg/dL ≤ serum Zn level <130 μg/dL were defined as group A (normal serum Zn level), those with 60 μg/dL ≤ serum Zn level < 80 μg/dl as group B (subclinical Zn deficiency) and those with serum Zn level < 60 μg/dL as group C (Zn deficiency). We retrospectively investigated the relationship between serum Zn level and baseline data, and the impact of serum Zn classification on survival compared with the C–P classification or the ALBI grading system. In addition, factors associated with overall survival (OS) were studied in the univariate and multivariate analyses. 

This study protocol was in compliance with the 1975 Helsinki Declaration, and was approved by the institutional review board in the Hyogo college of medicine (approval no. 2082). All personal information was protected during data collection. 

### 2.4. Statistical Analyses

In continuous variables, the statistical comparison among groups was performed by Student’s *t* test, analysis of variance (ANOVA), Mann–Whitney U test, Kruskal–Wallis test or Pearson’s correlation r, as appropriate. In the univariate analyses of factors associated with OS, the median value for each parameter was selected in order to divide the study population into two groups, which was then treated as nominal variables in the univariate analysis. Parameters with *p* < 0.01 in the univariate analysis were entered into the multivariate Cox hazard model. We made survival curves by the Kaplan–Meier method and compared them in the log-rank test. The Akaike information criterion (AIC) with each evaluation method was calculated in order to compare the OS. A lower AIC value suggests better prognostic ability. Data were presented as the median value with interquartile range (IQR). The significance level in the analysis was *p* < 0.05 using the statistical analysis software (JMP 14 (SAS Institute Inc., Cary, NC, USA)).

## 3. Results

### 3.1. Baseline Characteristics

**Figure 1:**
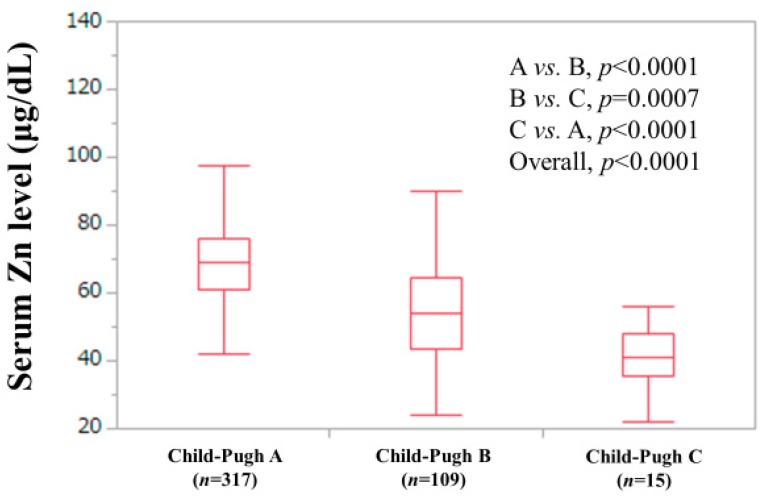
Serum Zn level according to the Child–Pugh classification.

**Figure 2:**
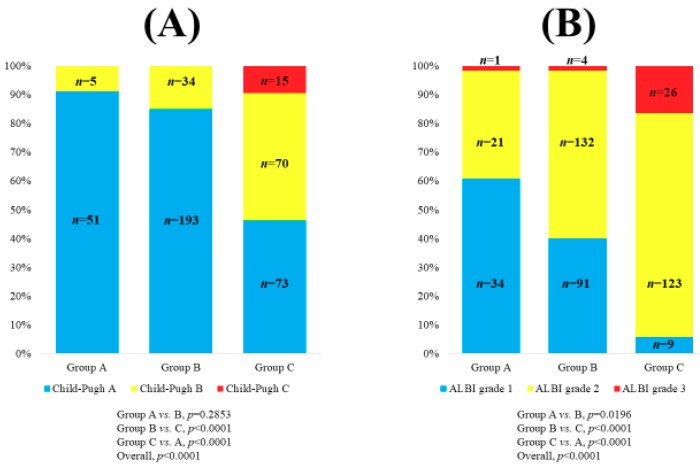
The number and distribution of the Child–Pugh classification in groups A, B and C (**A**) and ALBI grade in groups A, B and C (**B**). Patients with 80 μg/dL ≤ serum Zn level <130 μg/dL were defined as group A (normal serum Zn level), those with 60 μg/dL ≤ serum Zn level < 80 μg/dL as group B (subclinical Zn deficiency) and those with serum Zn level < 60 μg/dL as group C (Zn deficiency).

Demographic and clinical characteristics of the analyzed subjects (*n* = 441) were demonstrated in Table 1. The study cohort included 239 males and 202 females with the median age (IQR) of 66 (59.5, 72.5) years. The median (IQR) follow-up duration was 4.4 years (3.1, 7.5 years). The median (IQR) serum Zn level was 65 μg/dL (54, 74.4 μg/dL). There were 56 patients (12.7%) in group A, 227 patients (51.5%) in group B and 158 patients (35.8%) in group C. With regard to the C–P classification and liver disease etiologies, patients were predominantly C–P A (317/441, 71.9%) and HCV (253/441, 57.4%). The median (IQR) serum Zn level in patients with C–P A, B and C was 69 μg/dL (61, 76 μg/dL), 54 μg/dL (43.5, 64.45 μg/dL) and 41.1 μg/dL (35.5, 48.2 μg/dL) (*p* values: *p* < 0.0001 in C–P A vs. B, *p* = 0.0007 in C–P B vs. C and *p* < 0.0001 in C–P A vs. C; overall *p* value < 0.0001) (Figure 1). There were 134 patients (30.4%) with ALBI grade 1, 276 patients (62.6%) with ALBI grade 2 and 31 patients (7.0%) with ALBI grade 3. The proportion of patients with Child–Pugh classification A, B and C was well stratified among groups A, B and C (*p* values: *p* = 0.2853 in group A vs. B, *p*< 0.0001 in group B vs. C and *p* < 0.0001 in group A vs. C; overall *p* value <0.0001) (Figure 2A). Likewise, the proportion of patients with ALBI grade 1, 2 and 3 was well stratified among groups A, B and C (*p* values: *p* = 0.0196 in group A vs. B, *p*< 0.0001 in group B vs. C and *p* < 0.0001 in group A vs. C; overall *p* value < 0.0001) (Figure 2B).

### 3.2. Cumulative OS Rates According to Serum Zn Level Classification 

**Figure 3:**
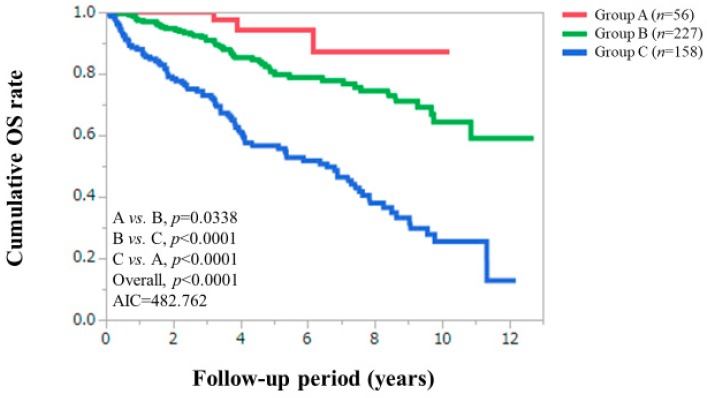
Cumulative overall survival according to the serum Zn classification proposed by the Japanese society of clinical nutrition. AIC, Akaike information criterion.

OS was our primary outcome measure. The 3, 5, 7 and 10 year cumulative OS rates were 100%, 94.57%, 87.29% and 87.29%, respectively, in patients in group A, 90.93%, 80.63%, 77.81% and 64.33%, respectively, in patients in group B, and 72.97%, 56.66%, 46.42% and 25.48%, respectively, in patients in group C (*p* values: *p* = 0.0338 in group A vs. B, *p* < 0.0001 in group B vs. C and *p* < 0.0001 in group A vs. C; overall *p* value < 0.0001) (Figure 3). 

### 3.3. Comparison of Prognostic Impact among C–P Classification, ALBI Grade and Zn Classification System

**Figure 4:**
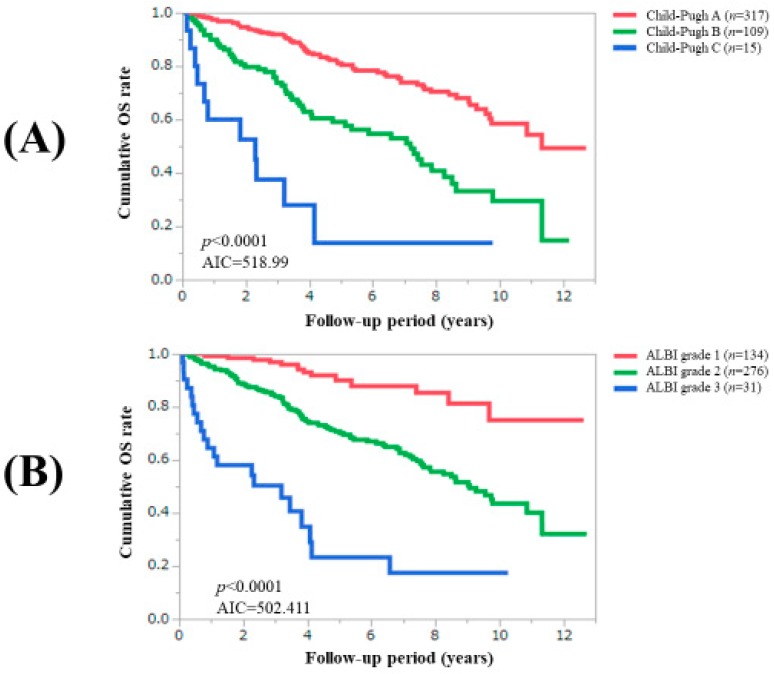
Cumulative overall survival according to the Child–Pugh classification (**A**) and ALBI grade (**B**). ALBI, albumin–bilirubin.

We compared predictive accuracy among three prognostic models for all cases. OS was well stratified by the C–P classification, ALBI grade and Zn classification system (*p* values, all <0.0001). The AIC value for survival by the Zn classification system was the lowest among three prognostic models (AIC: 518.99 in the C–P classification, 502.411 in ALBI grade and 482.762 in the Zn classification system) (Figure 3 and Figure 4).

### 3.4. Causes of Death According to Serum Zn Level Classification 

In group A, during the observation period, three deaths (5.4%) were observed. The causes and number of deaths included hepatic failure in two patients and advanced HCC status in one patient. In group B, during the observation period, 49 deaths (21.6%) were observed. The causes and number of deaths included hepatic failure in 31 patients, advanced HCC status in seven patients and other causes in 11 patients. In group C, during the observation period, 89 deaths (56.3%) were observed. The causes and number of deaths included hepatic failure in 62 patients, advanced HCC status in 18 patients and other causes in nine patients.

### 3.5. Serum Ammonia Level According to Serum Zn Level Classification 

**Figure 5:**
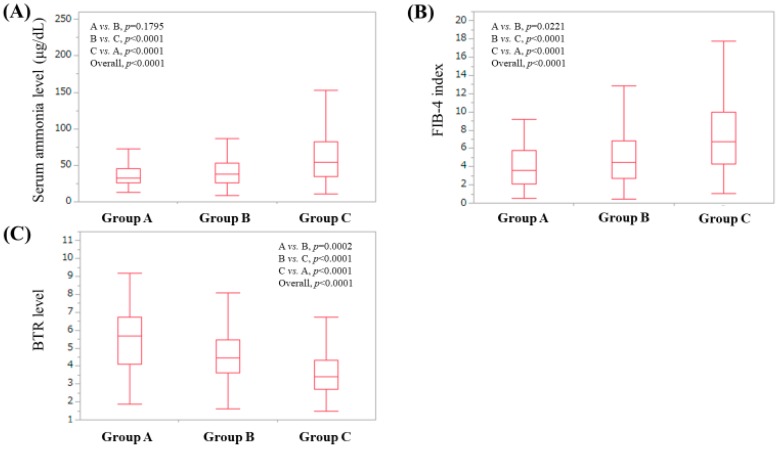
(**A**) Serum ammonia level according to the serum Zn classification proposed by the Japanese society of clinical nutrition. (**B**) The FIB-4 index according to the serum Zn classification proposed by the Japanese society of clinical nutrition. (**C**) Branched-chain amino acid to tyrosine ratio (BTR) according to the serum Zn classification proposed by the Japanese society of clinical nutrition. BTR, Branched-chain amino acid to tyrosine ratio.

The median (IQR) serum ammonia level in patients in group A, B and C was 33 μg/dL (26, 45 μg/dL), 38 μg/dL (26, 53 μg/dL) and 54.5 μg/dL (35, 82.75 μg/dL) (*p* values: *p* = 0.1795 in group A vs. B, *p* < 0.0001 in group B vs. C and *p* < 0.0001 in group A vs. C; overall *p* value < 0.0001) (Figure 5A).

### 3.6. FIB-4 Index According to Serum Zn Level Classification 

The median (IQR) serum FIB-4 index in patients in group A, B and C was 3.63 (2.0975, 5.7625), 4.47 (2.7, 6.79) and 6.745 (4.2675, 9.9475) (*p* values: *p* = 0.0221 in group A vs. B, *p* < 0.0001 in group B vs. C and *p* < 0.0001 in group A vs. C; overall *p* value <0.0001) (Figure 5B).

### 3.7. Branched-Chain Amino Acid to Tyrosine Ratio (BTR) Value According to Serum Zn Level Classification 

The median (IQR) serum BTR value in patients in group A, B and C was 5.665 (4.1175, 6.715), 4.45 (3.605, 5.45) and 3.415 (2.7, 4.3225) (*p* values: *p* = 0.0002 in group A vs. B, *p* < 0.0001 in group B vs. C and *p* < 0.0001 in group A vs. C; overall *p* value < 0.0001) (Figure 5C).

### 3.8. Correlation between Serum Zn Level and Baseline Data

**Figure 6:**
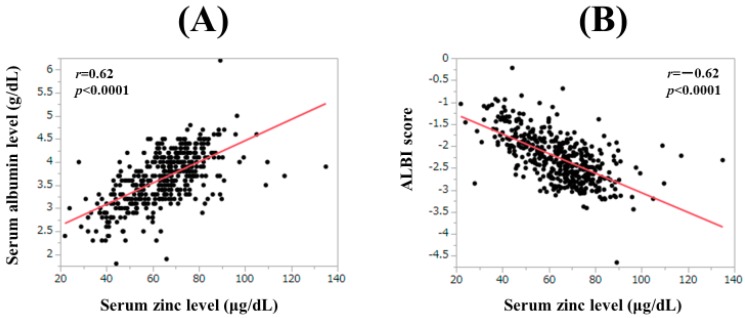
Correlation between serum Zn level and serum albumin level (**A**) and ALBI score (**B**).

The correlation coefficient between serum Zn level and baseline data was shown in Table 2. Serum albumin had the strongest positive correlation with serum Zn level (*r* = 0.62, *p* < 0.0001), while the ALBI score had the strongest negative correlation with serum Zn level (*r* = −0.62, *p* < 0.0001) (Figure 6A,B).

### 3.9. Uni- and Multivariate Analyses of Factors Linked to OS

The univariate analysis of factors linked to OS observed twelve factors with *p* < 0.01: age ≥66 years (*p* = 0.0010), the C–P classification (*p* < 0.0001), serum albumin level ≥3.7 g/dL (*p* < 0.0001), ALBI grade (*p* < 0.0001), MELD score ≥4.87 (*p* = 0.0003), PT ≥76.9% (*p* = 0.0002), platelet count ≥9.7 × 10^4^/mm^3^ (*p* = 0.0065), serum creatinine level ≥0.67 mg/dL (*p* = 0.0010), serum sodium level ≥140 mmol/L (*p* = 0.0070), serum Zn classification system (*p* < 0.0001), serum ammonia ≥40 μg/dL (*p* < 0.0001) and FIB-4 index ≥ 5.09 (*p* < 0.0001) (Table 3). Of these twelve factors, age, serum albumin, PT, serum creatinine and platelet count were not included in the multivariate analysis because age and platelet count are included in the FIB-4 index, and serum albumin and PT are included in the C–P classification or ALBI grade, and serum creatinine was included in the model for end-stage liver disease (MELD) score. Multivariate analysis for the remaining seven factors showed that serum ammonia ≥40 μg/dL (*p* < 0.0001), ALBI grade 3 (*p* = 0.0026, ALBI grade 1 as a reference), group B in the serum Zn classification system (*p* < 0.0001, group A as a reference) and group C in the serum Zn classification system (*p* < 0.0001, group A as a reference) were significant factors associated with OS (Table 4). Hazard ratios and 95% confidence intervals for these items are shown in Table 4.

## 4. Discussion

As mentioned earlier, JSCN defines serum Zn level < 60 μg/dL as Zn deficiency, 60 μg/dL ≤ serum Zn level < 80 μg/dL as subclinical Zn deficiency, and 80 μg/dL ≤ serum Zn level < 130 μg/dL as normal Zn range. However, the predictability of this classification system in LC patients has not been fully elucidated, which prompted us to perform the current analysis. In our results, in terms of OS, the AIC value of serum Zn classification by JSCN was the lowest among three assessment methods, and in the multivariate analyses of factors linked to OS, serum Zn classification by JSCN was an independent factor. Additionally, the C–P classification, ALBI grade, serum ammonia level, FIB-4 index and BTR were well stratified by this classification system. These results denoted that the serum Zn classification by JSCN can be useful for predicting clinical outcomes, hepatic function or ammonia clearance in LC patients. Kaplan–Meier curves demonstrated that maintaining serum Zn level >80 μg/dl may be ideal for the management of LC patients. In our data, 158 patients (35.8%) had Zn deficiency (serum Zn level < 60 μg/dL) at baseline and serum Zn level had the strong positive correlation with serum albumin level (*r* = 0.62, *p* < 0.0001), which were in agreement with previous data [33]. Possible causes for Zn deficiency in LC patients include disturbed Zn absorption from the digestive tract and increased Zn excretion in the urine [19,34]. In LC patients with Zn deficiency, Zn supplementation therapy may be beneficial, although investigation of the impact of Zn supplementation therapy on outcomes in LC patients was beyond the scope of this study. Serum Zn level was also well stratified among Child–Pugh A, B and C groups in the current analysis. Child–Pugh B or C patients have a higher prevalence of malnutrition compared with Child–Pugh A patients [1,2,3]. Thus, serum Zn level can be a surrogate marker for poor nutritional status in LC patients. 

Notably, the ALBI score had the strongest negative correlation with serum Zn level (*r* = −0.62, *p* < 0.0001) among baseline parameters in the current analysis. Recently, the ALBI grading system has been gaining popularity because of its convenience of use in clinical settings [35,36]. Close monitoring for serum Zn level may be required. While in the multivariate analysis, serum ammonia level was an independent predictor for OS. Hyperammonemia caused by advanced LC status can lead to hypermyostatinemia (myostatin is a negative regulator of muscle mass synthesis), and subsequent serious complications including sarcopenia, which may be linked to adverse outcomes [37,38]. Serum ammonia lowering therapy may thus be essential for LC patients. Zn is involved in the function of urea cycle of the liver, and Zn deficiency causes hyperammonemia through impaired ammonia metabolic capacity due to the dysfunction of the urea cycle [33]. The significant negative correlation between serum Zn level and serum ammonia level was noted (*r* = −0.34, *p* < 0.0001) in our results. 

Recently, the introduction of oral DAA agents has dramatically improved sustained virological response (SVR) rates in HCV therapy, providing SVR rates over 95% with shorter HCV treatment duration and a good safety profile [39,40]. In addition, several NAs for HBV-related LC patients are currently available with a favorable therapeutic response [41]. In group A, the 3 year OS rate was 100%. One possible reason for these results was that there were a lot of HCV-related LC or HBV-related LC patients with favorable treatment response for antiviral therapies during the follow-up period, which may be linked to the high survival rate in group A. On the other hand, the mechanism for the serum zinc level decline in HCV-related LC is presumed to involve the non-structural proteins (NS)3 and NS5A of HCV [41,42]. NS3 is a Zn-containing enzyme, and NS5A is a Zn metalloprotein [42,43]. In this study, 147 LC patients (58.1%) achieved SVR during the follow-up period. Elevated serum albumin level and serum Zn level can be found in HCV patients with SVR [44]. Nevertheless, the serum Zn classification system had the lowest AIC among three assessment methods in our HCV patients (AIC: 281.598 in the C–P classification, 270.884 in the ALBI grade and 240.175 in the Zn classification system, not shown in the results section), denoting the robustness of the serum Zn classification system by JSCN. 

We must acknowledge several limitations in the present study. Firstly, this is a single center observational study with a retrospective study design and the usefulness of the serum Zn classification should be verified in other independent cohorts. Secondly, the number of our C–P C patients was pretty small compared to that of C–P A or B patients, thus creating bias. Thirdly, our study population was limited to LC patients without HCC; whether the serum Zn classification could be extrapolated to HCC patients or non-LC patients needs future research. Fourthly, Zn supplementation therapy or other nutritional therapy during the follow-up period were not included in this analysis. Caution must be therefore taken in the interpretation of our data. However, our study results suggest that the serum Zn classification by JSCN can be a useful grading system in LC patients. 

## 5. Conclusions

In conclusion, the serum Zn classification proposed by JSCN appears to be helpful for estimating prognosis in LC patients. 

## Figures and Tables

**Table 1:** Baseline characteristics (*n* = 441).

Variables	All Cases (*n* = 441)
Age (years)	66 (59.5, 72.5)
Gender, male/female	239 (54.2)/202 (45.8)
Cause of liver disease, HBV/HCV/others	46 (10.4)/253 (57.4)/142 (32.2)
Child–Pugh classification, A/B/C	317 (71.9)/109 (24.7)/15 (3.4)
MELD score	4.87 (2.20, 7.36)
Ascites, yes/no	59 (13.4)/382 (86.6)
Hepatic encephalopathy, yes/no	6 (1.4)/435 (98.6)
Body mass index (kg/m^2^)	23.1 (20.5, 25.7)
Total bilirubin (mg/dL)	1.0 (0.7, 1.5)
Serum albumin (g/dL)	3.7 (3.2, 4.05)
ALBI score	−2.31 (−2.67, −1.92)
ALBI grade, 1/2/3	134 (30.4)/276 (62.6)/31 (7.0)
Prothrombin time (%)	76.9 (66.0, 86.3)
Platelet count (×10^4^/mm^3^)	9.7 (6.9, 13.6)
AST (IU/l)	39 (27, 60)
ALT (IU/l)	30.5 (20, 49)
Total cholesterol (mg/dL)	151.5 (129.25, 177)
Fasting blood glucose (mg/dL)	102 (92.25, 118)
Serum creatinine (mg/dL)	0.67 (0.57, 0.79)
Serum sodium (mmol/L)	140 (138, 141)
Serum Zn level (μg/dL)	65 (54, 74.4)
Serum Zn level classification, <130, ≥80 μg/dL/<80, ≥60 μg/dL/<60 μg/dL	56 (12.7)/227 (51.5)/158 (35.8)
Branched-chain amino acid to tyrosine ratio	4.13 (3.235, 5.375)
Serum ammonia (µg/dL)	40 (29, 62)
FIB-4 index	5.09 (3.18, 7.77)

Data are expressed as number (%) or median value (interquartile range). HBV, hepatitis B virus; HCV, hepatitis C virus; MELD, model for end-stage liver disease; ALBI, albumin–bilirubin; AST, aspartate aminotransferase; ALT, alanine aminotransferase; Zn, zinc

**Table 2:** Correlation between serum Zn level and baseline data.

Variables	*r*	*p* Value
Age	−0.10	0.0420
Body mass index	−0.04	0.4015
Total bilirubin	−0.27	<0.0001
Serum albumin	0.62	<0.0001
ALBI score	−0.62	<0.0001
Prothrombin time	0.35	<0.0001
Platelet count	0.14	0.0031
AST	−0.27	<0.0001
ALT	−0.13	0.0050
Total cholesterol	0.19	<0.0001
Fasting blood glucose	−0.06	0.1909
Serum creatinine	−0.02	0.6695
Serum sodium	0.27	<0.0001
BTR	0.42	<0.0001
Serum ammonia	−0.34	<0.0001
MELD score	−0.29	<0.0001
FIB-4 index	−0.32	<0.0001

ALBI, albumin–bilirubin; AST, aspartate aminotransferase; ALT, alanine aminotransferase; BTR, branched-chain amino acid to tyrosine ratio; MELD, model for end-stage liver disease.

**Table 3:** Univariate analyses of factors linked to overall survival.

Variables	*n*	*p* Value
Age ≥ 66 years, yes/no	234/207	0.0010
Gender, male/female	239/202	0.1605
HBV/HCV/others	46/253/142	0.0140
Child–Pugh classification, A/B/C	317/109/15	<0.0001
Body mass index ≥ 23.1 kg/m^2^, yes/no	221/220	0.0366
Total bilirubin ≥ 1.0 mg/dL, yes/no	253/188	0.0177
Serum albumin ≥ 3.7 g/dL, yes/no	232/209	<0.0001
ALBI grade, 1/2/3	134/276/31	<0.0001
MELD score ≥ 4.87, yes/no	220/221	0.0003
Prothrombin time ≥ 76.9%, yes/no	221/220	0.0020
Platelet count ≥ 9.7 × 10^4^/mm^3^, yes/no	223/216	0.0065
AST ≥ 39 IU/l, yes/no	224/216	0.4417
ALT ≥ 30.5 IU/l, yes/no	220/220	0.3604
Total cholesterol ≥ 151.5 mg/dL, yes/no	216/216	0.0969
Fasting blood glucose ≥ 102 mg/dL, yes/no	226/206	0.5393
Serum creatinine ≥ 0.67 mg/dL, yes/no	223/218	0.0010
Serum sodium ≥ 140 mmol/L, yes/no	266/175	0.0070
Zn, <130, ≥ 80 μg/dL/<80, ≥ 60 μg/dL/<60 μg/dL	56/227/158	<0.0001
BTR ≥ 4.13, yes/no	220/217	0.0149
Serum ammonia ≥ 40 μg/dL, yes/no	227/199	<0.0001
FIB-4 index ≥ 5.09, yes/no	220/219	<0.0001

HBV, hepatitis B virus; HCV, hepatitis C virus; ALBI, albumin–bilirubin; MELD, model for end-stage liver disease; AST, aspartate aminotransferase; ALT, alanine aminotransferase; Zn, zinc; BTR, branched-chain amino acid to tyrosine ratio.

**Table 4:** Multivariate analyses of factors linked to overall survival.

Variables	Multivariate Analysis		
	HR	95% CI	*p* Value
Serum ammonia level ≥ 40 µg/dL	1.995	1.540–2.583	<0.0001
FIB-4 index ≥ 5.09	1.210	0.926–1.582	0.1622
MELD score ≥ 4.87	1.139	0.880–1.473	0.3234
Serum sodium ≥ 140 mmol/L	0.870	0.672–1.126	0.2894
ALBI grade			
Grade 1	1.000	Reference	
Grade 2	1.127	0.524–2.429	0.7600
Grade 3	1.540	1.163–2.041	0.0026
Child–Pugh classification			
Child–Pugh A	1.000	Reference	
Child–Pugh B	1.042	0.715–1.519	0.8309
Child–Pugh C	2.409	0.791–7.339	0.1219
Serum Zn classification by JSCN			
Group A	1.000	Reference	
Group B	2.003	1.438–2.790	<0.0001
Group C	2.533	1.640–3.911	<0.0001

HR, hazard ratio; CI, confidence interval; MELD, model for end-stage liver disease; ALBI, albumin–bilirubin; Zn, zinc; JSCN, Japanese society of clinical nutrition.
